# Enteropathy‐associated T‐cell lymphoma: A population‐based cohort study on incidence, treatment, and outcome in the Netherlands

**DOI:** 10.1002/jha2.1049

**Published:** 2024-11-29

**Authors:** Frederik O. Meeuwes, Mirian Brink, Wouter J. Plattel, Joost S. P. Vermaat, Marie José Kersten, Mariëlle Wondergem, Otto Visser, Marjolein W. M. van der Poel, Rimke Oostvogels, F. J. Sherida H. Woei‐A‐Jin, Lara Böhmer, Tjeerd J. F. Snijders, Gerwin A. Huls, Marcel Nijland

**Affiliations:** ^1^ Department of Hematology University Medical Center Groningen Groningen the Netherlands; ^2^ Department of Hematology Medisch Spectrum Twente Enschede the Netherlands; ^3^ Department of Research and Development Netherlands Comprehensive Cancer Organization (IKNL) Utrecht the Netherlands; ^4^ Department of Hematology Leiden University Medical Center Leiden the Netherlands; ^5^ Department of Hematology Amsterdam University Medical Center, Cancer Center Amsterdam University of Amsterdam Amsterdam the Netherlands; ^6^ Department of Hematology Isala Hospital Zwolle the Netherlands; ^7^ Department of Internal Medicine Division of Hematology GROW School for Oncology and Developmental Biology, Maastricht University Medical Center Maastricht the Netherlands; ^8^ Department of Hematology University Medical Center Utrecht Utrecht the Netherlands; ^9^ Department of General Medical Oncology University Hospitals Leuven Leuven Belgium; ^10^ Department of Hematology Haga Teaching Hospital the Hague the Netherlands

**Keywords:** ASCT, EATL, enteropathy‐associated T‐cell lymphoma, etoposide, outcome

## Abstract

**Introduction:**

Enteropathy‐associated T‐cell lymphoma (EATL) is a peripheral T‐cell lymphoma (PTCL) with a poor prognosis. Cyclophosphamide, doxorubicin, vincristine, and prednisone (CHOP) with or without etoposide consolidated by autologous stem cell transplantation (ASCT) are recommended for fit PTCL patients. The role of etoposide and ASCT in EATL is unclear.

**Methods:**

This study reports the incidence, treatment, and outcome of EATL patients using the Netherlands Cancer Registry, with nationwide coverage of >95%.

**Results:**

All patients diagnosed in 1989–2021 (*n* = 351, 77% treated) were identified (median age 67 years, 56% male, 50% limited stage). Time period analysis assessed trends in primary therapy and overall survival (OS). Treatment included chemotherapy (CT) (34%), surgery (18%), surgery and CT (19%) or CT followed by ASCT (7%). The 5‐year OS for treated patients with limited versus advanced stage was 19% and 9% respectively. The 2‐year OS improved over time (21%–33%, *p = *0.06). Surgery only (hazard ratio [HR] 2.16; 95% confidence interval [CI] 1.55–3.01, *p <* 0.01) and advanced‐stage disease (HR 1.67; 95% CI 1.25–2.23, *p =* 0.01) were predictors of poor prognosis. ASCT (HR 0.31; 95% CI 0.18–0.56) was associated with improved OS.

**Conclusion:**

There was no statistical difference in OS between patients treated with or without etoposide. Current first‐line treatment is ineffective.

## 1 INTRODUCTION

Enteropathy‐associated T‐cell lymphoma (EATL) is a rare, aggressive peripheral T‐cell lymphoma (PTCL). It is classified into two subtypes; EATL (previously known as EATL type I) and monomorphic epitheliotropic intestinal T‐cell lymphoma (MEITL) (previously known as EATL type II) [1, 2]. EATL is a rare complication of celiac disease (CD) [3, 4, 5, 6]. MEITL is more commonly seen in Asian populations and is not clearly associated with CD [1, 7–9]. EATL comprises 5% of the PTCLs and 10%–16% of the intestinal lymphomas [1, 9–11].

When abnormal intraepithelial lymphocyte expansion is present, refractory CD (RCD) is diagnosed and the consequent risk of developing EATL is 60%–80% [3–5, 12, 13]. Primary EATL develops ‘de novo’, often presenting with obstruction or perforation, and a diagnosis of CD is made concomitantly. Secondary EATL occurs in known (R)CD cases and patients often have a poor performance status due to prolonged malnutrition [12, 13, 14].

The prognosis of EATL is poor, with a median 5‐year overall survival (OS) of 0–59% and a median OS of 7 months. In EATL patients with previously diagnosed RCD, the 5‐year OS is only 0–8% [1, 3, 6, 13–19]. MEITL and EATL have a similarly poor prognosis [9, 11].

There is no universally accepted standard therapy for EATL. Randomized clinical trials addressing first‐line treatment modalities in EATL patients have not been performed and are considered not feasible due to the rarity of the disease. Patients are generally treated with cyclophosphamide, doxorubicin, vincristine, and prednisone—either with or without etoposide (CHO(E)P). In other PTCL subtypes, studies show that adding etoposide to CHOP is not beneficial, except in ALK + ALCL [20, 21, 22, 23, 24, 25]. In PTCL, autologous stem cell transplant (ASCT) is associated with improved survival in fit patients in the majority of studies [25, 26, 27, 28, 29]. Furthermore, combined modality treatment (CMT), where an abbreviated chemotherapy regimen is combined with radiotherapy, shows superior outcomes in stage 1(E) PTCL [30, 31, 32]. In EATL however, the role of etoposide and ASCT is still unclear as well as the role of CMT in limited‐stage disease. Our nationwide population‐based cohort study aims to describe the incidence and evaluate the various first‐line treatment regimens and outcomes of patients with EATL diagnosed in the Netherlands between 1989 and 2021, in particular patients with limited‐stage disease.

## 2 MATERIAL AND METHODS

### 2.1 Registry

The nationwide population‐based Netherlands Cancer Registry (NCR) is maintained and hosted by the Netherlands Comprehensive Cancer Organization (IKNL) and has nationwide coverage of at least 95% of all malignancies since 1989 [33]. The NCR relies on comprehensive case notification through the Nationwide Histopathology and Cytopathology Data Network and the Nationwide Registry of Hospital Discharges (i.e., inpatient and outpatient discharges). Information on dates of birth and diagnosis, sex, topography and morphology, hospital type of diagnosis, and first‐line therapy is routinely recorded by trained registrars of the NCR through retrospective medical records review. Information on the exact therapeutic regimen was registered in the NCR for patients diagnosed as of January 1, 2014. Information on the last known vital status for all patients (i.e., alive, dead, or emigration) is obtained through annual linkage with the Nationwide Population Registries Network which holds vital statistics on all residents of the Netherlands.

### 2.2 Study population

All newly diagnosed patients ≥ 18 years with EATL who were diagnosed between 1989 and 2021 were identified in the Netherlands Cancer Registry (NCR), using the International Coding System of Disease – Oncology (ICD‐O) of the World Health Organization (WHO), morphology codes 9702/3 and 9717/3 which includes both EATL and MEITL. Patients who were diagnosed post‐mortem were excluded (*n* = 2). Survival follow‐up was available through February 1, 2023. The NCR records first‐line treatment initiated within 12 months post‐diagnosis. Patients were categorized according to the treatment regimen, that is, surgery, chemotherapy (CT), CT followed by autologous stem cell transplantation (ASCT), surgery and CT, or no treatment. Calendar period analysis was conducted to assess trends in primary therapy and OS over time, choosing three periods of (approximately) 10 years (1989‐1999, 2000–2010, and 2010–2021). The staging was done according to the Ann Arbor/Lugano classification for staging of lymphomas, where limited stage was defined as stage I/II and advanced stage disease was defined as stage III/IV. According to the Central Committee on Research involving Human Subjects, this type of observational study does not require approval from an ethics committee in the Netherlands. The Privacy Review Board of the NCR approved the use of anonymous data for this study.

### 2.3 Statistical analysis

Descriptive statistics were used to present patient and treatment characteristics per calendar period, and according to stage. The primary endpoint was OS, defined as the time between EATL diagnosis and all‐cause death. The Kaplan‐Meier method served to estimate OS, and the log‐rank test to examine differences in survival distributions. OS was calculated for the total cohort as well as for the three calendar periods. Furthermore, OS was calculated according to stage. Subsequently, only patients who received first‐line treatment were analyzed, again for the total cohort, as well as for the three calendar periods and according to stage. Then, the impact of age, sex, and treatment modality (ASCT as a time‐varying covariable) on the risk of mortality was evaluated using uni‐ and multivariable Cox proportional hazard regression analysis. The results from the Cox regression analyses produce hazard ratios (HRs) with associated 95% confidence intervals (CIs). The proportional hazard assumption was tested based on the Schoenfeld residuals. Covariables were introduced in the regression models with a backward selection method. The final model was accomplished when the *p*‐value for the covariables was below 0.05. A sub‐analysis was performed on the cohort of 2014–2021, since exact information on therapy was available from 2014 onward, describing the various treatment regimens. For those treated with chemotherapy, a sensitivity analysis was performed to assess the outcome of patients treated with CHOP versus those treated with CHOEP, excluding those receiving other chemotherapeutic regimens. Patients alive were censored on February 1, 2023. Overall, a *p*‐value below 0.05 was considered statistically significant. All analyses were performed using STATA/SE 17.1 (StataCorp LP).

## 3 RESULTS

### 3.1 Patient characteristics

Between 1989 and 2021, 5367 patients with PTCL were registered in the NCR, of whom 351 (7%) patients were diagnosed with EATL. Over time, there was an increase in average annual incidence from 6.4 diagnoses per year in 1989–1999 to 14.9 diagnoses per year in 2011–2021. The median age of these patients was 67 years (range 34–90 years) and 56% was male. Limited stage was more common than advanced stage disease (50% vs. 39%, 11% undetermined). Over time, the proportion of patients with advanced‐stage disease increased from 19% in 1989–1999 to 54% in 2010–2021, whereas the proportion of patients with a limited stage and an unknown stage decreased (Table 1).

**TABLE 1:** Baseline characteristics of patients with enteropathy‐associated T‐cell lymphoma (EATL), according to period of diagnosis.

	1989–1999		2000–2010		2011–2021		Total cohort	
	*n* = 64		*n* = 138		*n* = 149		*n* = 351	
	No.	%	No.	%	No.	%	No	%
**Male sex**	36	56	77	56	83	56	196	56
**Age at diagnosis**								
Median age	64 (41–86)		65 (37–90)		71 (34–85)		67 (34–90)	
<65 years	33	52	61	44	44	30	138	39
65–74 years	17	26	39	28	29	19	85	24
≥75 years	14	22	38	28	76	51	128	36
**Disease stage**								
Limited	38	59	79	57	58	39	175	50
Advanced	12	19	46	33	80	54	138	39
Unknown	14	22	13	9	11	7	38	11
**Median follow‐up, months (range)**								
All patients	7.2 (0.07–299.4)		3.7 (0.03–273.0)		6.6 (0.03–133.9)		5.2 (0.03–299.4)	
Number of patients alive at date of censuring	1		6		27		34	

### 3.2 Treatment

**FIGURE 1:**
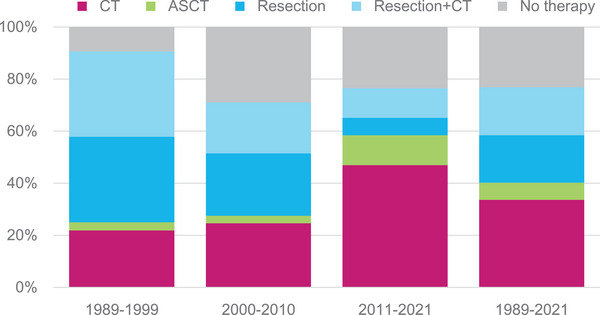
Treatment modalities of patients with enteropathy‐associated T‐cell lymphoma (EATL), according to time period.

Treatment consisted of CT (34%), surgery (18%), surgery and CT (19%), CT followed by ASCT (7%), or no treatment (23%). Two patients received radiotherapy, one in the ASCT group and one in the surgery + CT group. Over time, the percentage of patients treated with CT increased from 22% to 47%, whereas the percentage of patients who were treated by means of surgical resection only decreased from 33% to 6% (Figure 1). Fewer patients have been treated using the combination of surgery and chemotherapy as well (decrease from 33% to 11%). In total, the use of CT either as monotherapy or combined with other treatment modalities increased from 58% to 70% whereas the use of surgery, either as monotherapy or combined with other treatment modalities, decreased from 66% to 18%. In the group of the undetermined stage, 45% of patients did not receive treatment at all versus 19% and 22% in the limited and advanced stage disease patients, respectively (Figure 1).

**FIGURE 2:**
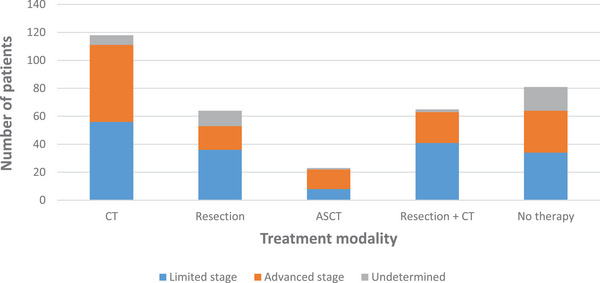
Treatment modalities of patients with enteropathy‐associated T‐cell lymphoma (EATL), according to the stage.

Comparing treatment regimens of limited stage and advanced stage, treatment consisted of CT in 32% versus 40%, surgery in 21% versus 12%, surgery and CT in 23% versus 16% and CT followed by ASCT in 5% versus 9%; no treatment was initiated in 19% versus 22% (Figure 2).

From 2014 onward, more detailed information on treatment regimens was available. Between 2014 and 2021, 106 patients were diagnosed with EATL (limited stage *n* = 40 [37%]; advanced stage *n* = 59 [56%]; undetermined stage *n* = 7 [7%]). In this cohort, 78 patients (74%) were treated with CT of whom 58% received less than six cycles and 42% 6–8 cycles. Of the patients with limited‐stage disease who were treated with chemotherapy, sixteen received CHOP (55%) and nine received CHOEP (31%). For patients with advanced disease who were treated with chemotherapy, 29 received CHOP (60%), and 15 received CHOEP (31%). Five patients with limited‐stage disease and six patients with advanced‐stage disease underwent ASCT (Table 2). Only eight patients received a regimen other than CHOP or CHOEP in first‐line, such as cladribine, COP, CEOP, or prednisone/chlorambucil/etoposide/lomustine (PECC).

**TABLE 2:** Uni‐ and multivariable analysis for risk of mortality of patients with enteropathy‐associated T‐cell lymphoma (EATL) receiving first‐line treatment.

	Limited stage (*n* = 40)		Advanced stage (*n* = 59)		Unknown stage (*n* = 7)	
	No.	%	No.	%	No.	%
**CHOEP**	**9**	***23***	**15**	***25***	**–**	***–***
< 6 cycles	4	*44*	9	*60*	–	*–*
6 cycles	5	*56*	6	*40*	–	*–*
6–8 cycles	–	–	–	–	–	*–*
ASCT	3	*33*	3	*20*	–	*–*
**CHOP**	**16**	***40***	**29**	***49***	**1**	***14***
< 6 cycles	9		16		1	
6 cycles	6		10		–	*–*
6–8 cycles	1		3		–	*–*
ASCT	2	*13*	3	*10*	–	*–*
**Other**	**4**	***10***	**4**	***7***	**–**	***–***
ASCT	–	*–*	–	*–*	–	*–*
No systemic therapy	11	*27*	11	*19*	6	*86*

Abbreviations: ASCT, autologous stemcell transplant, CHOEP, cyclophosphamide, doxorubicin, etoposide, vincristine, and prednisone, CHOP, cyclophosphamide, doxorubicin, vincristine, and prednisone.

### 3.3 Outcome

**FIGURE 3:**
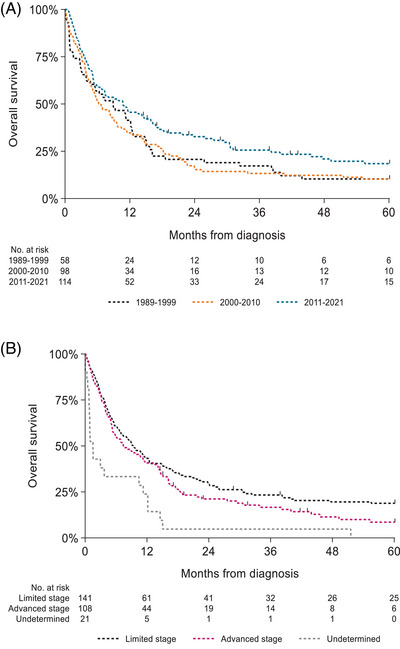
(A) Overall survival of all patients receiving first‐line treatment according to time period. (B) Overall survival according to the stage of all patients receiving first‐line treatment.

The 2‐year and 5‐year OS among the 351 patients was 19% and 11%, respectively. The median OS was 5.2 months for the total cohort, 6.3 months for the limited stage, and 5.2 months for the advanced stage. Patients who did not receive treatment had a median OS of 0.8 months. Therefore, further analyses were focused on the group of 270 patients (77%) who received first‐line treatment. With a median follow‐up time of 8.3 months, the 2‐year and 5‐year OS were 24% and 14%, respectively. The 2‐year OS improved from 21% in 1989–1999 to 33% in 2011–2021, whereas the 5‐year OS improved from 10% to 18% in the same calendar periods (Figure 3A). The 6‐month, 2‐year, and 5‐year OS for patients with limited‐stage disease was 60%, 29%, and 19% (median OS 9.3 months) and for patients with advanced stage 55%, 21% and 9% (median OS 7.4 months), respectively (Figure 3B). Those patients who underwent ASCT had a 2‐year OS of 69% and a median OS of 20 months. In multivariable analysis, surgery only (hazard ratio [HR] 1.51; 95% confidence interval [CI] 1.04–2.17, *p = *0.03), and advanced stage disease (HR 1.43; 95% CI 1.10–1.85, *p <* *0.01*) were independent predictors of poor prognosis, whereas ASCT (HR 0.38; 95% CI 0.21–0.69) and a diagnosis of EATL between 2011 and 2021 (HR 0.68; 95% CI 0.47–1.00) were associated with lower risk of mortality (Table 3). In a sensitivity analysis of the 106 patients diagnosed between 2014 and 2021, no statistical difference was found in OS between patients treated with CHOP or CHOEP.

**TABLE 3:** Chemotherapy (CT) regimen, when used, according to the stage for patients diagnosed 2014–2021.

	Univariable			Multivariable		
	HR	95% CI	*p*	HR	95% CI	*p*
**Therapy**						
CT only	*1*	*Reference*		*1*	*Reference*	
Resection	1.63	1.15–2.29	<0.01	1.51	1.04–2.17	0.03
ASCT	0.37	0.20–0.67	<0.01	0.38	0.21–0.69	<0.01
Resection+CT	0.92	0.66–1.27	0.61	0.85	0.60–1.19	0.34
**Age**						
18–64	*1*	*Reference*				
65–70	1.26	0.93–1.69	0.13	–	–	
70+	1.37	1.04–1.80	0.03	–	–	
**Sex**						
Male	*1*	*Reference*				
Female	1.18	0.93–1.49	0.19	–	–	
**Stage**						
Limited	*1*	*Reference*		*1*	*Reference*	
Advanced	1.22	0.96–1.55	0.10	1.43	1.10–1.85	<0.01
**Period**						
1989–1999	*1*	*Reference*				
2000–2010	1.33	0.95–1.87	0.10	1.04	0.73–1.46	0.84
2011–2021	0.89	0.63–1.25	0.49	0.68	0.47–1.00	0.05

Abbreviations: ASCT, autologous stemcell transplant, CI, confidence interval; CT, chemotherapy; HR, hazard ratio.

## 4 DISCUSSION

Over a span of 32 years (1989–2021), a total of 351 patients were diagnosed with EATL. There was an increase in EATL diagnoses over time, which is in line with expectations due to both the overall rise in cancer diagnoses and the rising incidence of CD in the Netherlands. The proportion of patients with limited and unknown stages decreased significantly over time, which may be due to the increased use of FDG PET‐scanning and therefore more accurate disease staging whereas an increase in disease awareness might have prevented patients from succumbing to the disease undiagnosed. On the other hand, the median age of patients increased from 64 to 71 years. During this timeframe, the use of CT, either combined or as monotherapy, increased whereas the use of surgery decreased. The increased survival over time was associated with an increased use of CT. The vast majority of patients who were treated with CT received either CHOP or CHOEP, few patients underwent ASCT whereas CMT (in a limited stage) was not used at all. The long‐term outcome remains poor. We observed a favorable outcome for the small number of patients (*n* = 22) receiving consolidative ASCT. The addition of etoposide to CHOP was not associated with improved outcomes.

In the Netherlands, the median OS was even worse than the 6–8 months mentioned in previous studies [1, 3, 6, 13–19]. However, the outcome has slightly improved from 5‐year OS of 10% in 1989–1999 to 18% in 2011–2021 (excluding untreated patients). This might be due to the increased use of ASCT in recent years. There was a statistically significant – albeit not clinically meaningful – difference between the outcome of patients with limited‐stage disease and those with advanced‐stage disease, confirming that stage seems to have little impact on outcome in EATL [1, 3, 11, 13].

In a cohort of patients with stage I(E) anaplastic large cell lymphoma (ALCL), angioimmunoblastic T‐cell lymphoma (AITL), and PTCL not otherwise specified (PTCL NOS), 28% were treated with CMT [32]. In the current study, CMT was not applied at all in patients with limited‐stage EATL. Whether this is due to disease characteristics, unfamiliarity with CMT, the poor prognosis and the presumed need for more chemotherapy, or foreseen technical difficulties planning radiotherapy for small bowel lesions, is unknown. With current magnetic resonance imaging‐guided radiation techniques, we hypothesize that CMT might be possible in limited‐stage EATL and this is an interesting subject for future research. A potential problem would be how to define the radiation field when the lymphoma has already vanished due to chemotherapy; this could however easily be solved by treating the patient with radiotherapy first, followed by three courses of CHOP chemotherapy.

In those patients treated from 2014 onward, CHOP was the most commonly used regimen. Adding etoposide to CHOP did not improve the outcome, however, the number of patients in both groups is small. The outcomes of patients with EATL treated with CHOEP in earlier studies are mixed due to different study designs—namely with or without consolidative ASCT—small numbers of patients and selection bias [34, 35]. Of those patients diagnosed and treated from 2014 onward, only 14% have received ASCT, highlighting that many are unfit for intensive treatment or have refractory disease. However, those patients who underwent ASCT had a favorable prognosis with a 5‐year OS of 69%, although the numbers are very small. In other study populations, those patients who had undergone ASCT had a similarly superior prognosis, with a 4‐year OS of 59% and a 5‐year OS of 60% respectively [12, 18].

Randomized clinical trials are rare in PTCL in general and, to our knowledge, non‐existent in EATL. There is however sparse data from phase 2 studies. The strategy where one course of CHOP is followed by the IVE/MTX‐regimen (ifosfamide/etoposide/epirubicin alternated with intermediate‐dose MTX [3 g/m^2^]) followed by high‐dose therapy – mostly carmustine/etoposide/cytarabine/melphalan (BEAM) – and ASCT has shown promising results in a case series of both 6 and 26 patients, where the 5‐year progression‐free survival (PFS) and OS were 52% and 60% respectively in the latter study [12, 37]. In daily practice, this regimen has not been universally adapted for the first‐line treatment of EATL patients, although it bears potential. The ECHELON‐2 trial showed that the anti‐CD30 antibody‐drug conjugate brentuximab vedotin (BV) + CHP was superior to CHOP in PTCL, however only three patients are a promising treatment option. A French study investigating CHP‐BV followed by consolidation with ASCT as a frontline treatment for patients with EATL (*n* = 14) showed an overall response rate of 79% and those who responded all underwent ASCT. The 2‐year PFS and OS were 63% and 68%, respectively [38].

Mutations in the JAK1‐STAT3 and NF‐κ B pathways are found in the majority of patients with RCD. In vitro, JAK‐inhibitors ruxolitinib and abocritinib and the proteasome inhibitor bortezomib reduced proliferation and induced apoptosis in RCD cell lines. Therefore, JAK inhibitors and proteasome inhibitors seem attractive for further investigation [4].

The main strengths of our study include the use of a nationwide population‐based cancer registry with comprehensive data available on first‐line treatment in a homogeneous patient population. Furthermore, this is the largest cohort of EATL patients published up to now. Limitations of our study include a lack of information on comorbidities. Moreover, a central review by a specialist pathologist of the tumor biopsies was unavailable due to the nature of this study. Information on whether underlying CD was already known or diagnosed concurrently with EATL or not at all, is unfortunately unavailable. Detailed information on tumor and treatment characteristics was available from 2014 onward. Despite its limitations, cancer registries remain the standard for cancer surveillance activities and for population‐based analysis of treatment outcomes. With limited data being available on patients with EATL, these data are highly valuable.

## 5 CONCLUSION

The outlook for patients diagnosed with EATL remains grim, marked by a significant early mortality rate and a notable absence of survival plateau. With a median OS of 5 months and a 5‐year OS rate of merely 11%, challenges persist across all stages of the disease. The current first‐line treatment is ineffective and underscores the urgent need for the development of innovative therapeutic strategies. For those that do achieve remission on induction therapy, ASCT seems to improve outcomes, warranting future research.

## AUTHOR CONTRIBUTIONS

Frederik O. Meeuwes, Marcel Nijland, and Mirian Brink designed the study. Mirian Brink collected the data. Mirian Brink, Marcel Nijland, and Frederik O. Meeuwes analyzed the data. Frederik O. Meeuwes, Mirian Brink, and Marcel Nijland wrote the paper. All authors revised the manuscript and accepted its final version.

## CONFLICT OF INTEREST STATEMENT

The authors declare no conflict of interest.

## ETHICS STATEMENT

The authors have confirmed ethical approval statement is not needed for this submission.

## PATIENT CONSENT STATEMENT

The authors have confirmed patient consent statement is not needed for this submission.

## CLINICAL TRIAL REGISTRATION

The authors have confirmed clinical trial registration is not needed for this submission.

## Data Availability

Data will not be available to share due to Dutch laws and regulations.
